# Skin microbiome profile of healthy Cameroonians and Japanese

**DOI:** 10.1038/s41598-022-05244-5

**Published:** 2022-01-25

**Authors:** Kazuhiro Ogai, Benderli Christine Nana, Yukie Michelle Lloyd, John Paul Arios, Boonyanudh Jiyarom, Honore Awanakam, Livo Forgu Esemu, Aki Hori, Ayaka Matsuoka, Firzan Nainu, Rosette Megnekou, Rose Gana Fomban Leke, Gabriel Loni Ekali, Shigefumi Okamoto, Takayuki Kuraishi

**Affiliations:** 1 AI Hospital/Macro Signal Dynamics Research and Development Center (ai@ku), Institute of Medical, Pharmaceutical and Health Sciences, Kanazawa University, Kanazawa, Japan; 2 Biotechnology Center, University of Yaoundé I, Yaoundé, Cameroon; 3 Department of Animal Biology and Physiology of the Faculty of Science, University of Yaoundé I, Yaoundé, Cameroon; 4 Department of Tropical Medicine, Medical Microbiology and Pharmacology, John A. Burns School of Medicine, University of Hawaii at Manoa, Hawaii, USA; 5 Institute of Medical Research and Medicinal Plant Studies, Ministry of Scientific Research and Innovation, Yaoundé, Cameroon; 6 Laboratory of Host Defense and Responses, Faculty of Pharmacy, Institute of Medical, Pharmaceutical and Health Sciences, Kanazawa University, Kakuma-machi, Kanazawa, Ishikawa 920-1192 Japan; 7 Faculty of Health Sciences, Institute of Medical, Pharmaceutical and Health Sciences, Kanazawa University, Kanazawa, Japan; 8 Department of Pharmacy, Faculty of Pharmacy, Hasanuddin University, Makassar, Indonesia; 9 Advanced Health Care Science Research Unit, Institute for Frontier Science Initiative, Kanazawa University, 5-11-80 Kodatsuno, Kanazawa, Ishikawa 920-0942 Japan

**Keywords:** Metagenomics, Metagenomics, Microbiome, Policy and public health in microbiology

## Abstract

The commensal microbes of the skin have a significant impact on dermal physiology and pathophysiology. Racial and geographical differences in the skin microbiome are suggested and may play a role in the sensitivity to dermatological disorders, including infectious diseases. However, little is known about the skin microbiome profiles of people living in Central Africa, where severe tropical infectious diseases impose a burden on the inhabitants. This study provided the skin profiles of healthy Cameroonians in different body sites and compared them to healthy Japanese participants. The skin microbiome of Cameroonians was distinguishable from that of Japanese in all skin sites examined in this study. For example, *Micrococcus* was predominantly found in skin samples of Cameroonians but mostly absent in Japanese skin samples. Instead, the relative abundance of *Cutibacterium* species was significantly higher in healthy Japanese. Principal coordinate analysis of beta diversity showed that the skin microbiome of Cameroonians formed different clusters from Japanese, suggesting a substantial difference in the microbiome profiles between participants of both countries. In addition, the alpha diversity in skin microbes was higher in Cameroonians than Japanese participants. These data may offer insights into the determinant factors responsible for the distinctness of the skin microbiome of people living in Central Africa and Asia.

## Introduction

Skin is one of the most crucial protective features available in humans. With diverse anatomical and physiological functions, starting from barrier function to thermostat regulation, the skin plays an essential role in a human’s life^1^. At present, the skin has been regarded as one of the largest microbial ecosystems found in the body, possibly due to direct contact and prolonged exposure to the dynamic external environment^2–7^. Indeed, the skin’s unique physiological and topographical features provide various distinct yet suitable niches for diverse types of microbial communities^8^. Although much focus has been given, the skin remains one of the less-charted territories in the field of microbiome investigation^9–13^. Nevertheless, the increase of sophisticated research tools, such as the utilization of next-generation sequencing and powerful bioinformatics applications, has opened the possibilities to carry out some exciting yet unexplored ideas in this field.

Skin microbial communities are influenced by many factors. Some of them depend on the interpersonal variation of host factors found in the human skin. Such variations may include a distinct set of topographical features and different temperatures, pH values, and sebum content^14^. These niche-specific physiological differences influence resident bacteria and fungi^9,10,15^; oily surfaces, such as the forehead, support lipophilic bacteria that differ from dry, low biomass sites, such as the forearm. The question arises: What bacteria must grow in some skin regions to have a healthy normal life? Solving this challenging puzzle would offer great relevance to the comprehensive study of the human microbiome.

Skin microbiome and dermatology-related studies are closely connected^9,10,14^. Some studies have suggested the role of the skin microbiome in developing certain dermatological disorders, such as acne, atopic dermatitis, psoriasis, rosacea, and seborrheic dermatitis^2^. Besides, dysbiosis of the skin microbiome has been implicated in the progressiveness of certain dermatological diseases. For example, leprosy and alteration of the skin microbiota composition in response to diseases and therapeutical interventions (such as systemic antimicrobials preparations) have been recently suggested^16^. To this end, it is interesting to see whether the composition of the skin microbiome in individuals of different countries or even different races will determine their susceptibilities to certain infectious diseases.

Several aspects, such as physiological and topographical features, have been suggested to influence the establishment of skin microbiome at individual levels^6,14,15^. Also, early reports have suggested that race^17^ and skin body sites^18^ are essential factors to consider in the skin microbiome profile. However, although the essentiality of these two factors is likely, information on them remains scarce. This study cataloged the skin profile of healthy Cameroonians and healthy Japanese in different body sites to support such an endeavor. Some studies have covered the skin microbiome of healthy Japanese^19–21^. However, to the authors’ knowledge, this study is the first to characterize the skin microbiome profile in three skin sites of healthy Cameroonians and compare the results to the ones obtained from healthy Japanese. This is the first comparative report describing the skin microbiome profile of people living in Central Africa (the west coast of the African continent) and the Asian population. These results may provide fundamental insights into the determinant factors responsible for the establishment of skin microbiota.

## Results

### Distinctive differences in the skin microbes between Cameroonians and Japanese

Dynamic variations in the human skin microbiome have been suggested to be associated with host factors (including age, diet, body sites, and sex) and environmental conditions (such as geographical location and climate)^2,5^. This study examined the relative abundance and prevalence of microbes in skin swabs collected from healthy participants of two different countries: Cameroon (n = 27) and Japan (n = 21). The demographic information was described in Table 1.

**Table 1:** Demographic data of the participants.

	Cameroon	Japan
*n*	27	21
Sex, *n*, female/male	20/7	12/9
Age, y, mean ± SD	31.2 ± 8.4	26.2 ± 4.1

SD, standard deviation.

**Figure 1:**
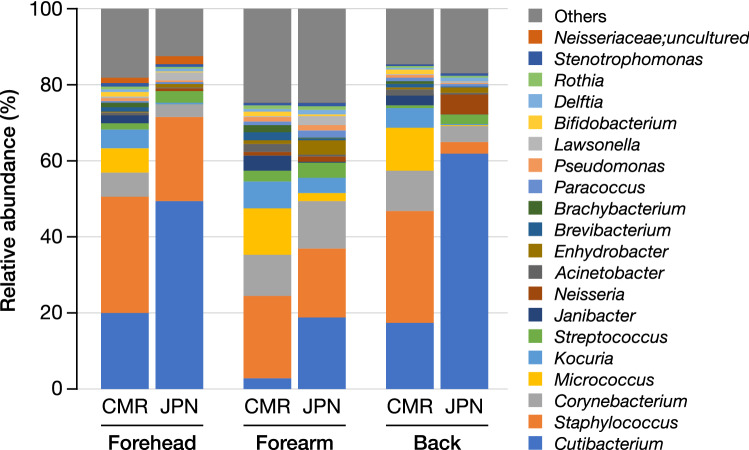
Top 20 skin microbiome at various positions between Cameroonian and Japanese participants. CMR, Cameroonian; JPN, Japanese.

Figure 1 shows a comparison of the skin microbiome compositions between healthy Cameroonians and healthy Japanese in the three skin sites: forehead, right forearm, and the mid-upper back (Supplementary Fig. S1). The microbiome of the Cameroonian and Japanese skin samples can be assigned into more than 20 genera that mostly belonged in three phyla: Actinobacteria, Proteobacteria, and Firmicutes. At the genus level, most obtained sequences were *Cutibacterium*, *Staphylococcus*, *Micrococcus*, *Corynebacterium*, *Kocuria*, and, to a lesser extent, *Janibacter* and *Streptococcus* (Fig. 1 and Table 2)*.*

**Table 2:** Interracial comparison of microbial abundance in three different skin sites between Cameroonian and Japanese participants.

Genus	Forehead				Forearm				Back			
	Rel. abund. (%)		*p*-value	*q*-value	Rel. abund. (%)		*p*-value	*q*-value	Rel. abund. (%)		*p*-value	*q*-value
	CMR	JPN			CMR	JPN			CMR	JPN		
*Cutibacterium*	17.56	49.02	< 0.001	0.002	0.65	7.60	< 0.001	< 0.001	9.85	73.31	< 0.001	< 0.001
*Staphylococcus*	26.68	13.37	0.07	0.13	13.91	17.63	0.70	1.0	18.80	2.20	< 0.001	< 0.001
*Micrococcus*	4.39	0.03	< 0.001	< 0.001	8.56	0.06	< 0.001	< 0.001	7.66	0.04	< 0.001	< 0.001
*Corynebacterium*	4.75	2.05	0.002	0.01	8.51	15.75	1.0	1.0	8.36	1.67	0.002	0.004
*Kocuria*	2.42	0.06	< 0.001	< 0.001	3.88	0.75	0.01	0.02	1.96	0.05	< 0.001	< 0.001
*Janibacter*	0.53	0.00	< 0.001	< 0.001	0.74	0.02	< 0.001	< 0.001	0.50	0.01	< 0.001	< 0.001
*Streptococcus*	0.74	1.21	0.48	0.48	1.34	2.68	0.23	0.68	0.29	0.55	0.09	0.09

Rel. abund., relative abundance (median); CMR, Cameroonian; JPN, Japanese.

Of all, *Staphylococcus* was predominantly found in all three body sites of Cameroonians and Japanese. However, it is important to note that the relative abundance of *Staphylococcus* at the back of Japanese participants was lower than that of other skin sites examined in this study. In addition, although the *Cutibacterium* population was higher in all three skin sites of Japanese than Cameroonians, its presence was highly observed in samples recovered from sebaceous (forehead and back) sites, similar to the results of the previous study^8^.

Compared to their Japanese counterparts, there were some distinct features of the skin microbiome in the Cameroonian skin. *Micrococcus* was predominantly found in Cameroonian skin samples but mostly absent in Japanese skin samples. Likewise, further analysis indicated that *Kocuria* was present in all skin samples of healthy Cameroonians but observable at a low amount only in the forearm skin sample of healthy Japanese. In contrast, the low abundance of *Neisseria and Enhydrobacter* seemed more prevalent to be recovered from Japanese skin samples, mostly in the back and forearm, respectively, than that of Cameroonians. Overall, these findings implied the involvement of geographical- or race-related factors in the difference of skin microbiome of the two countries.

### Little effect of sex on the differences in skin microbiome between the two countries

Sexual dimorphism in microbiome composition has been suggested^22^. To investigate whether sexual dimorphism is a determinant in the difference of skin microbe composition observed in this study, skin samples collected at the forehead, forearm, and the back of healthy men and women participants from Cameroon and Japan were analyzed (Supplementary Fig. S2). A two-way (sex × country) analysis of variance revealed that the country difference had much more effects on the difference in the relative abundance of major skin microbiota than the sex difference, as indicated by the larger partial *η*^2^ index in the “country” term of the analysis, although a few genera on the specific area (e.g., *Cutibacterium* on the forehead and back skin) showed significant differences according to sex (Supplementary Table S1). In sum, a certain distinctiveness in the skin microbiome observed in participants from the two countries could scarcely be explained by sexual dimorphism.

### Distinguishable beta diversity and higher alpha diversity in Cameroonians

**Figure 2:**
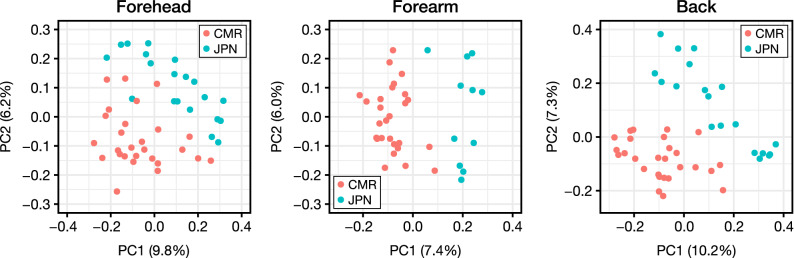
Beta diversity in reflection of the unweighted UniFrac distance. CMR, Cameroonian; JPN, Japanese; PC, principal coordinate.

We have shown that the composition of skin microbiome in the Cameroonian people were apparently different from that in the Japanese people. This finding was further confirmed by the beta diversity analysis using the unweighted UniFrac distance. The principal coordinate analysis of beta diversity showed that the skin samples obtained from healthy Cameroonian participants formed apparently different clusters from healthy Japanese participants (Fig. 2), suggesting the difference of microbiome profiles between the two countries.

**Figure 3:**
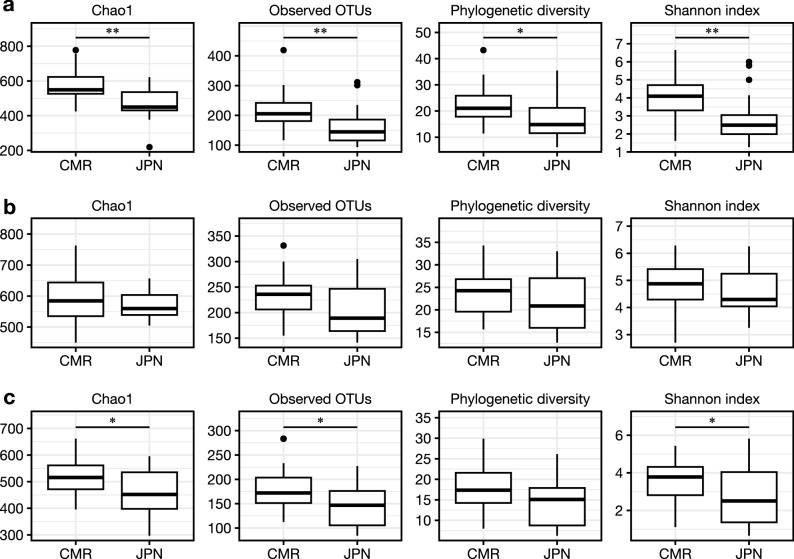
Alpha diversity of the forehead (A), forearm (B), and back (C) skin. CMR, Cameroonian; JPN, Japanese; OTU, operational taxonomic unit. *p < 0.05, **p < 0.01.

Figure 3 shows the alpha diversity metrics, namely Chao1, the number of the observed operational taxonomic units (OTUs), Faith’s phylogenetic diversity, and Shannon index, of the skin microbiome between healthy Cameroonians and healthy Japanese in the three skin sites: forehead (Fig. 3a), forearm (Fig. 3b), and the back (Fig. 3c). The species richness and diversity of Cameroonians were higher than those of Japanese counterparts, as suggested by the significant increase in Chao1, observed OTUs, and Shannon index values on the forehead and the back skin (Fig. 3a, c). However, most, if not all, alpha diversity indices showed no significant differences between both groups on the forearm skin (Fig. 3b).

## Discussion

This study analyzed the skin microbiome profile of healthy Cameroonians in three different body sites and compared them to healthy Japanese participants. Using the next generation sequencing (NGS) approach, whether geographical (to a greater extent, including racial difference) and topological features (three different skin sites: forehead, forearm, and the back) play distinctive roles in the human skin microbiome diversity was assessed. Consequently, the skin microbiome of Cameroonians was clearly distinguishable from that of Japanese individuals in all skin sites examined in this study.

This study identified a total collection of human skin microbiomes classified into more than 20 genera and spanned to at least three prominent phyla: Actinobacteria, Firmicutes, and Proteobacteria. Indeed, in agreement with this study, previous reports have demonstrated that the human skin microbiome is dominated by a similar microbiome profile^4,8,23,24^. Among the identified genera, *Cutibacterium*, *Staphylococcus*, *Micrococcus*, *Corynebacterium*, *Kocuria*, *Janibacter*, and *Streptococcus* were the most presented microbial genera on the skin of healthy participants from both countries (Fig. 1 and Table 2).

The relative abundance of *Cutibacterium* species (spp.) was significantly higher in healthy Japanese than healthy Cameroonians. This finding was in line with the skin microbiome studies in Asian population, which showed a high abundance of *Cutibacterium* spp. in healthy Chinese^25^, Korean^26^, and Japanese^19^. Three species of *Cutibacterium* (*C. avidum*, *C. acnes*, and *C. granulosum*) have been reported to colonize the skin surfaces of not only healthy individuals but also acne patients, possibly due to the attractiveness of these *Cutibacterium* species to the sebum-rich site of the skin^27^. This result implied that the Cameroonian skin environment might contain less sebum-rich ecological niches that support *Cutibacterium* spp. growth, although we do not have information regarding skin physiological functions due to inability to access to the equipment in Cameroon. Such a physiological assessment of the skin is warranted.

*Staphylococcus* was predominantly found in all three body sites of healthy Cameroonians and healthy Japanese. *Staphylococcus* spp. have been reported as the dominant and common bacterial colonizers of the skin^28^. This is in line with the result of this study in which the relative abundance of *Staphylococcus* spp. did not significantly different between Cameroonians and Japanese except on the back skin, likely due to much higher *Cutibacterium* spp. on the Japanese participants’ back skin (Fig. 1 and Table 2).

*Micrococcus* was predominantly found in skin samples of Cameroonians but mostly absent in Japanese skin samples. *Micrococcus* is a bacterial genus with a wide range of habitats, including dust and soil^29,30^, and has no difficulty growing in environments with little water or high-salt concentrations^31,32^. Likewise, *Kocuria* was abundantly present in all skin samples of healthy Cameroonians but only observable in the forearm skin samples of healthy Japanese. Although several species of *Kocuria* have been reported to inhabit the human skin and oral cavity^33^, *Kocuria* also has been reported to be widely spread in an environmental niche^34,35^. Taking these into account, the presence of *Micrococcus* and *Kocuria* in skin samples of Cameroonians might in part implicate plausible contact interaction of Cameroonians with the external natural ecological system, for example, farms, gardens, and beaches and/or dust-based environments that are plentiful in Cameroon on a regular basis. Several studies have also reported the effect of contact with soil, aquatic, and other environmental sources of microbiota on the skin microbiome^36–38^. However, due to the lack of accurate information on the lifestyle of the participants, we do not know the extent to which they interact with the natural environment. Further skin microbiome studies that take lifestyle into account are needed to show the influence of the external environment on the differences in the skin microbiome of the two countries.

The skin microbiome can be influenced by a number of external factors, such as lifestyle, hygiene, geographical and/or climate differences^39–41^. In addition, intrinsic factors such as aging, sex, and racial differences are also said to be responsible for skin microbial variability^26,39,41,42^. In this study, the Cameroonians and the Japanese people showed substantially different skin microbiome profiles; however, the question remains as to which factors may be relevant to such differences. A previous study by Harker, et al.^43^ revealed the link between axilla skin microbiome and a single nucleotide polymorphism of *ABCC11* gene whose genotype varies according to racial differences^44^. In these studies, the G/G genotype which is common in African people led to significantly more *Staphylococcus* on the axillary skin, compared to Asian people who generally carry the A/A and G/A genotypes. These findings could possibly explain higher, albeit not significantly, *Staphylococcus* on the forearm and back skin of Cameroonian people in this study (Table 2). However, the study also showed that the G/G genotype was related to significantly less *Corynebacterium*, and *Micrococcus* was not affected by the *ABCC11* genotypes, both of which contradict the results of this study (Table 2). Although the genetic variation is a possible contributor to the race-microbiome relationship^45,46^, we do not have information on genetic background of the participants and thus the relationship among race, genetic variation, and microbiome cannot be further discussed in this study. Genome-wide association studies^46^ can be beneficial to investigate the involvement of genetic factors in the distinct skin microbiome between Cameroon and Japan.

Overall, the results suggested the presence of a diverse microbiome on the human skin. Although microbial diversity is distinguishable and similar species tend to reside in the same part of the skin of healthy subjects from both countries, the abundance of specific species is, to a larger extent, country (environment)-dependent and, to a lesser extent, skin site-dependent. With its limitations, the data suggested the possible relationship between the relative abundance of certain microbes with the geographical and topological features of the human skin. The inclusion of such information in a comprehensive analysis of skin microbes in healthy subjects may provide insights that would be beneficial in the discovery of safe and sound dermatological therapeutics to precisely cure and manage skin diseases.

## Methods

### Ethical considerations

This study was approved by the National Ethics Committee of Cameroon (approval no. 2018/06/1045/CE/CNERSH/SP), the hospital where the research was conducted, and the Medical Ethics Committee of Kanazawa University, where the NGS was performed (approval no. 894). All research was performed in accordance with the Declaration of Helsinki. Before inclusion, all participants were informed by a written document about the research, and written informed consent was obtained from all participants.

### Study design and settings

This was a part of a cross-sectional study conducted in a human immunodeficiency virus (HIV)-clinic of the Efoulan District Hospital in Yaoundé, Cameroon, and in a single university in Japan. The hospital was selected for an existing collaboration between the hospital and the university research center. Prior to recruitment of participants, we first made a tentative inclusion list of possible candidates for the study with the inclusion criteria of being aged 21–65 years. Each candidate received an explanation of this study by the researcher, including the following request. The candidates were requested not to (1) take a bath or shower or (2) use emollients/creams on the sampling site, after midnight of the day of sampling. Pregnant women, those who had apparent skin disorders (e.g., atopic dermatitis, psoriasis, and Kaposi sarcoma) or other systemic diseases (e.g., HIV infection), those who did not consent to the study, and those who did not follow the instruction requested by the researchers were excluded.

### Sample collection

Skin swabs were collected as described previously^47^. In brief, Puritan HydraFlock Sterile Flocked Swab (25–3306-H; Puritan Medical Products Co., ME, USA) was presoaked in normal saline (S5815; Teknova, CA, USA) with 0.1% Tween 20 (28353-14; Nacalai Tesque, Inc., Kyoto, Japan) solution. Swabbing was then performed in a 5 × 5 cm^2^ on designated positions. After swabbing, each swab head was cut and placed in a 1.5 mL microcentrifuge tube, carried in a cooler box with ice to prevent degradation of the samples, and stored at − 30 °C until DNA extraction.

### DNA extraction

DNA from the swab head was performed as described previously^47,48^. Briefly, the swab heads were processed with the Kaneka Easy DNA Extraction Kit version 2 (KN-T110005; Kaneka Corp., Tokyo, Japan), followed by the enzymatic DNA extraction process with QIAamp DNA Mini Kit (51,304; Qiagen N.V., Venlo, The Netherlands). The extracted DNA samples were stored at − 30 °C until NGS preparation.

### Controls

The swab without skin swabbing served as a negative control, and the ZymoBIOMICS Microbial Community Standard (D6300; Zymo Research Corp., Irvine, CA, USA) was used as a positive control (Supplementary Fig. 3). Little overlap between the observed skin microbiome (Fig. 1 and Table 2) and the negative control (Supplementary Fig. 3a) ruled out the possibility of contamination from the experimental materials (e.g., swabs and kits). The closeness of the theoretical and observed compositions of the positive control, especially the effective detection of gram-positive, skin-related *Staphylococcus*, implied the successful extraction and analysis of bacterial DNA (Supplementary Fig. 3b).

### NGS

The extracted DNA was dedicated to the NGS analysis as described previously^48^. The DNA samples were first quantified for the copy number of the 16S ribosomal RNA (rRNA) gene for normalization with a quality check. The same amount of the 16S rRNA gene from each sample was then used to amplify the V3-V4 hypervariable region^48–50^. After indexing polymerase chain reaction with the Nextera XT Index Kit version 2 (FC-131-2001 to 2004; Illumina, Inc., San Diego, CA, USA), the resultant library was loaded onto the MiSeq System (SY-410-1003; Illumina) with MiSeq Reagent Kit version 3, 600 cycles (MS-102-3003; Illumina), and 15% PhiX Control v3 (FC-110-3001; Illumina) spike-in.

### Sequence analysis

The raw fastq sequences were first quality-filtered by sickle^51^ and chimera-eliminated by usearch^52,53^. The high-quality, chimera-free sequences were then fed into the Qiime software version 1.9.1^54^. For the calculation of the OTUs, a 97% threshold was used. The Silva database^55^ version 132 was used for taxonomic assignment. Beta diversity was calculated based on the unweighted UniFrac distance followed by principal coordinate analysis. Alpha diversity indices [Chao1, observed OTUs, Faith’s phylogenetic diversity, and Shannon index] were calculated by the “alpha_rarefaction.py” command of Qiime1. The samples were rarefied at 3000 depth of sequences, and the samples with less than 3000 sequences were discarded.

### Statistical analysis

The data were expressed as means ± standard deviation, medians [interquartile range (IQR)], or *n* (%) where appropriate. The boxplot denotes the 25th, 50th, and 75th percentile boxes with 25th percentile − 1.5 × IQR to 75th percentile + 1.5 × IQR whiskers. For categorical variables, Fisher’s exact test was used. For continuous variables, Welch’s *t*-test or Mann–Whitney *U* test was used for two-group comparison where appropriate. A two-way analysis of variance with partial *η*^2^ values of effect size was used to take into account the two factors of sex and country differences in relative abundance. For multiple comparisons of bacterial abundance, Benjamini-Hochberg’s false discovery rate control was employed. The corrected *p*-values were denoted hereafter as *q*-values. *p*- or *q*-values < 0.05 was considered statistically significant. All statistical analyses were performed using R statistical software^56^ version 3.9.2.

## Supplementary Information

Supplementary Information.

## Data Availability

All raw sequences of the NGS analysis were deposited in DNA Data Bank of Japan (DDBJ; accession number is DRA011596).
