# Supplementary material for: Skin microbiome profile of healthy Cameroonians and Japanese
Source: Sci Rep. 2022 Jan 25;12:1364. doi: 10.1038/s41598-022-05244-5 (PMC8789912; doi:10.1038/s41598-022-05244-5)
Supplement: Supplementary file 1 — Supplementary Information. [file 41598_2022_5244_MOESM1_ESM.pdf]

**Skin microbiome profile of healthy Cameroonians and Japanese**

Kazuhiro Ogai[1], Benderli Christine Nana[2,3], Yukie Michelle Lloyd[4], John Paul Arios[4], Boonyanudh Jiyarom[4], Honore Awanakam[2], Livo Forgu Esemu[2,5], Aki Hori[6], Ayaka Matsuoka[7], Firzan Nainu[6,8], Rosette Megnekou[2,3], Rose Gana Fomban Leke[2,5], Gabriel Loni Ekali[2], Shigefumi Okamoto[7,9,*] and Takayuki Kuraishi[6,*]

[1] AI Hospital/Macro Signal Dynamics Research and Development Center (ai@ku), Institute of Medical, Pharmaceutical and Health Sciences, Kanazawa University, Japan

[2] Biotechnology Center, University of Yaoundé I, Cameroon

[3] Department of Animal Biology and Physiology of the Faculty of Science, University of Yaoundé I, Cameroon

[4] Department of Tropical Medicine, Medical Microbiology and Pharmacology, John A. Burns School of Medicine, University of Hawaii at Manoa, USA

[5] Institute of Medical Research and Medicinal Plant Studies, University of Yaoundé I, Cameroon

[6] Faculty of Pharmacy, Institute of Medical, Pharmaceutical and Health Sciences, Kanazawa University, Japan

[7] Faculty of Health Sciences, Institute of Medical, Pharmaceutical and Health Sciences, Kanazawa University, Japan

[8] Department of Pharmacy, Faculty of Pharmacy, Hasanuddin University, Indonesia

[9] Advanced Health Care Science Research Unit, Institute for Frontier Science Initiative, Kanazawa University

**SUPPLEMENTARY INFORMATION**

- Supplementary Figure S1
- Supplementary Figure S2
- Supplementary Figure S3
- Supplementary Table S1

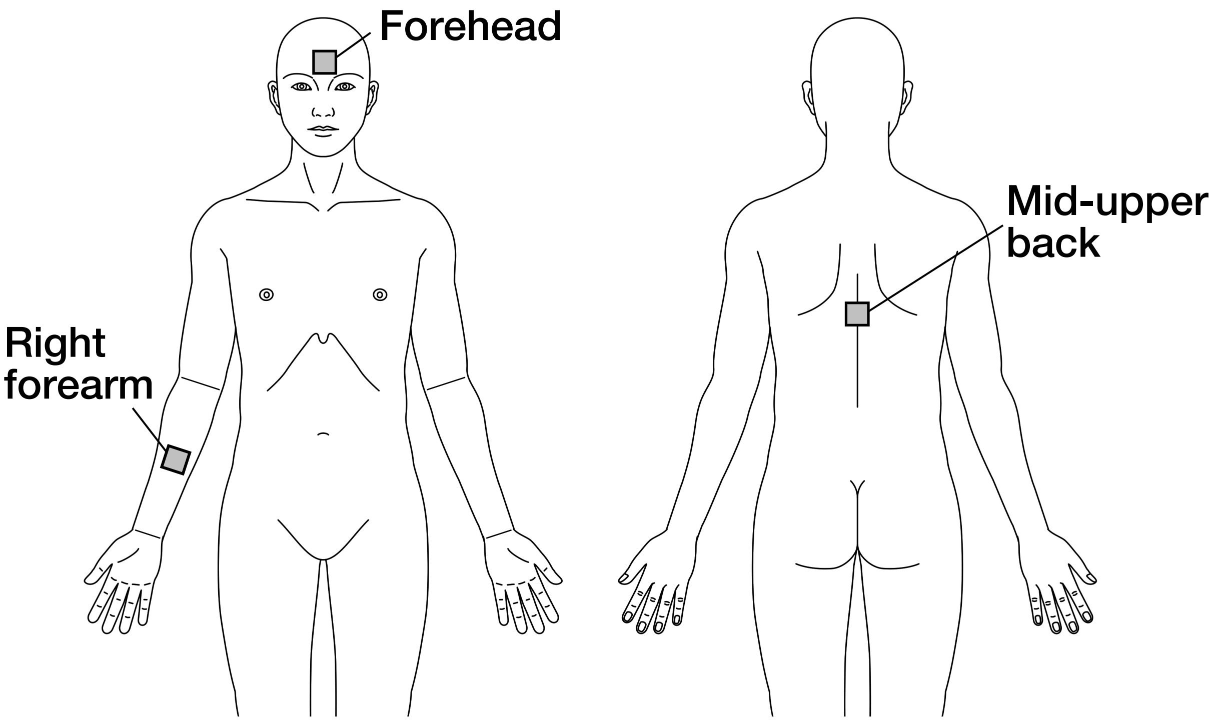


**Supplementary Figure S1**. Position of sampling.

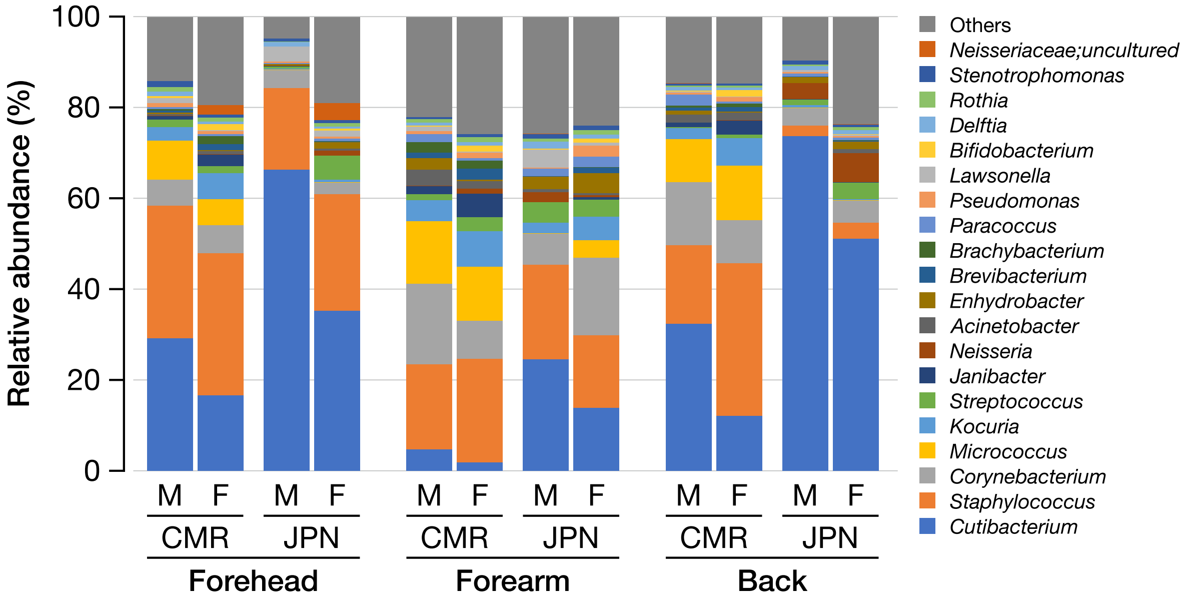


**Supplementary Figure S2**. Top 20 skin microbiome at various positions between Cameroonian and Japanese participants stratified by sex. CMR, Cameroonian; JPN, Japanese; M, male; F, female.

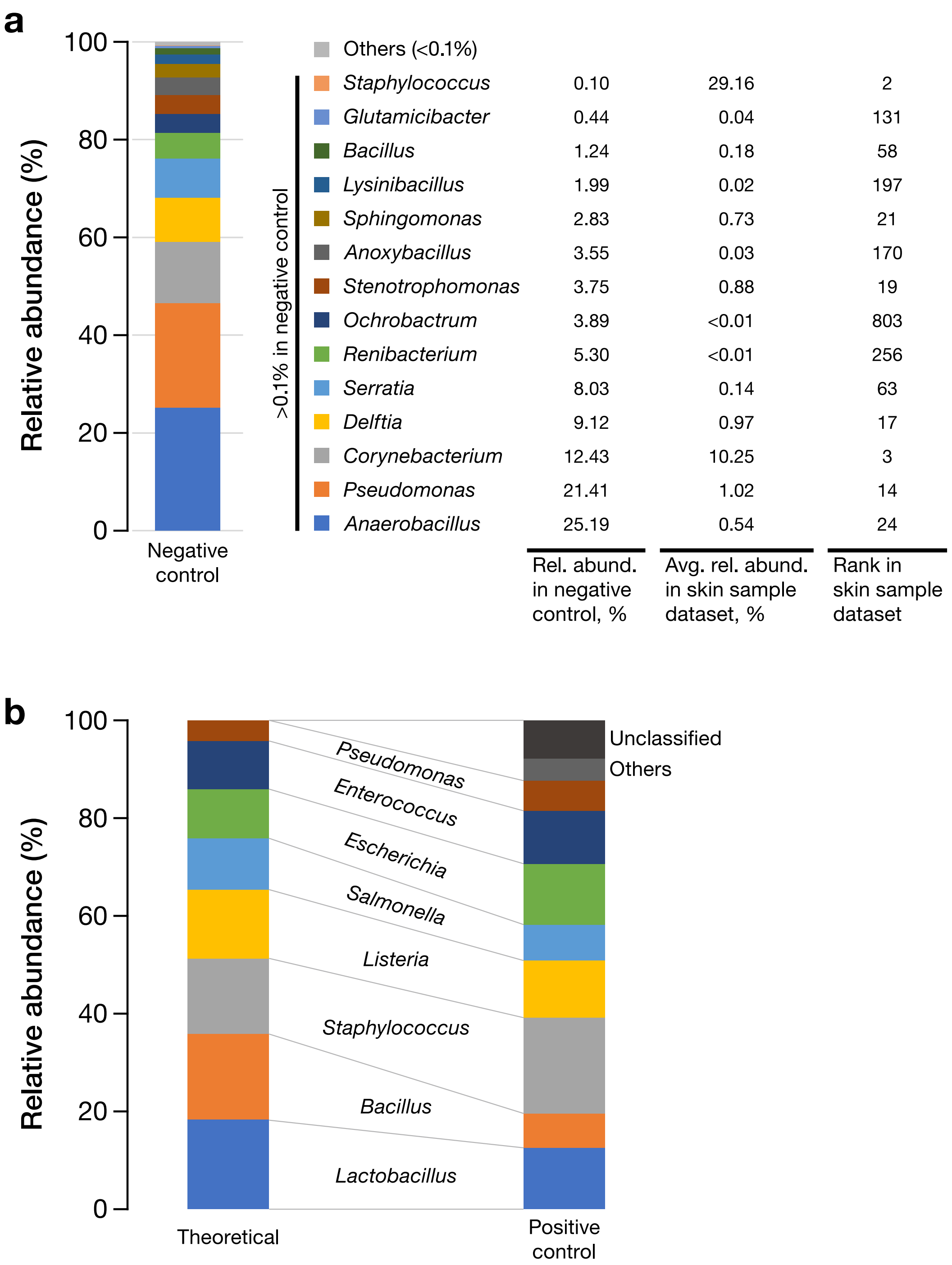


**Supplementary Figure S3**. Negative (**a**) and positive (**b**) experimental controls. The negative control was prepared by processing a swab without skin swabbing, and the positive control was prepared by processing the ZymoBIOMICS Microbial Community Standard (D6300; Zymo Research Corp., Irvine, CA, USA), both along with the other skin swabs which gave the results of the "skin sample dataset."

Rel. abund., relative abundance; avg., average.

**Supplementary Table S1**. Two-way (sex × country) analysis of variance with effect size for relative abundance of each genus in Table 1

| | | **Forehead** | | | **Forearm** | | | **Back** | | |
|---|---|---|---|---|---|---|---|---|---|---|
| **Genus** | **Term** | ***F* value** | ***p*-value** | **partial $\eta^2$** | ***F* value** | ***p*-value** | **partial $\eta^2$** | ***F* value** | ***p*-value** | **partial $\eta^2$** |
| *Cutibacterium* | sex | 9.58 | 0.003 | 0.182 | 3.20 | 0.08 | 0.086 | 7.07 | 0.011 | 0.144 |
| | country | 15.66 | <0.001 | **0.267** | 17.68 | <0.001 | **0.342** | 24.81 | <0.001 | **0.371** |
| | sex:country | 1.73 | 0.20 | 0.039 | 1.04 | 0.31 | 0.030 | 0.02 | 0.89 | 0.000 |
| *Staphylococcus* | sex | 0.61 | 0.44 | 0.014 | 0.00 | 0.95 | 0.000 | 4.16 | 0.048 | 0.090 |
| | country | 1.81 | 0.19 | **0.040** | 0.21 | 0.65 | 0.006 | 17.29 | <0.001 | **0.292** |
| | sex:country | 0.21 | 0.65 | 0.005 | 0.73 | 0.40 | **0.021** | 5.37 | 0.025 | 0.113 |
| *Micrococcus* | sex | 0.62 | 0.44 | 0.014 | 0.09 | 0.77 | 0.003 | 0.16 | 0.69 | 0.004 |
| | country | 15.10 | <0.001 | **0.260** | 10.38 | 0.0028 | **0.234** | 11.16 | 0.0018 | **0.210** |
| | sex:country | 0.68 | 0.41 | 0.016 | 0.71 | 0.40 | 0.021 | 0.15 | 0.70 | 0.004 |
| *Corynebacterium* | sex | 0.10 | 0.75 | 0.002 | 0.01 | 0.91 | 0.000 | 0.66 | 0.42 | 0.016 |
| | country | 30.45 | <0.001 | **0.415** | 0.10 | 0.76 | 0.003 | 11.41 | 0.0016 | **0.214** |
| | sex:country | 0.02 | 0.89 | 0.000 | 8.89 | 0.0053 | **0.207** | 1.53 | 0.22 | 0.035 |
| *Kocuria* | sex | 1.00 | 0.32 | 0.023 | 0.75 | 0.39 | **0.022** | 0.67 | 0.42 | 0.016 |
| | country | 6.00 | 0.018 | **0.122** | 0.50 | 0.48 | 0.015 | 3.68 | 0.062 | **0.081** |
| | sex:country | 0.56 | 0.46 | 0.013 | 0.00 | 0.95 | 0.000 | 0.92 | 0.34 | 0.021 |
| *Janibacter* | sex | 1.32 | 0.26 | 0.030 | 0.68 | 0.41 | 0.020 | 0.84 | 0.36 | 0.020 |
| | country | 4.85 | 0.033 | **0.101** | 1.72 | 0.20 | **0.048** | 2.82 | 0.10 | **0.063** |
| | sex:country | 1.47 | 0.23 | 0.033 | 0.36 | 0.55 | 0.011 | 0.76 | 0.39 | 0.018 |
| *Streptococcus* | sex | 5.27 | 0.027 | 0.109 | 0.18 | 0.68 | 0.005 | 3.27 | 0.078 | 0.072 |
| | country | 1.59 | 0.21 | 0.036 | 1.96 | 0.17 | **0.055** | 6.50 | 0.015 | **0.134** |
| | sex:country | 5.64 | 0.02 | **0.116** | 0.89 | 0.35 | 0.026 | 1.26 | 0.27 | 0.029 |

Term: sex, male vs female; country, Japan vs Cameroon; sex:country, interaction of sex and country.

Partial $\eta^2$, effect size; bold numbers represent the highest partial $\eta^2$ among the comparison (sex, country, and sex:country within the same genus and position).
